# Bactericidal and Virucidal Activities of Biogenic Metal-Based Nanoparticles: Advances and Perspectives

**DOI:** 10.3390/antibiotics10070783

**Published:** 2021-06-28

**Authors:** Gonzalo Tortella, Olga Rubilar, Paola Fincheira, Joana C. Pieretti, Paola Duran, Isabella M. Lourenço, Amedea B. Seabra

**Affiliations:** 1 Centro de Excelencia en Investigación Biotecnológica Aplicada al Medio Ambiente (CIBAMA), Facultad de Ingeniería y Ciencias, Universidad de La Frontera, Av. Francisco Salazar 01145, Temuco 4811230, Chile; olga.rubilar@ufrontera.cl (O.R.); p.fincheira01@ufromail.cl (P.F.); 2 Departamento de Ingeniería Química, Universidad de La Frontera, Av. Francisco Salazar 01145, Casilla 54-D, Temuco 4811230, Chile; 3 Center for Natural and Human Sciences, Universidade Federal do ABC, Santo André 09210-580, Brazil; joana.pieretti@ufabc.edu.br (J.C.P.); isabella.lourenco@ufabc.edu.br (I.M.L.); amedea.seabra@ufabc.edu.br (A.B.S.); 4 Biocontrol Research Laboratory, Scientific and Technological Bioresource Nucleus, Universidad de La Frontera, Temuco 4811230, Chile; paola.duran@ufrontera.cl

**Keywords:** antimicrobial nanoparticles, biogenic synthesis, metal nanoparticles, antibacterial, antivirus, nanobiotecnology

## Abstract

Much progress has been achieved in the preparation and application of engineered nanoparticles (NPs) in the field of medicine, mainly for antibacterial and antiviral applications. In the war against bacteria and viruses, besides traditional antibiotics and antiviral drugs, metal-based nanoparticles, such as silver (AgNPs), copper (CuNPs), copper oxides (CuO-NPs), iron oxide (FeO-NPs), zinc oxide (ZnO-NPs), and titanium oxide (TiO_2_-NPs) have been used as potent antimicrobial agents. These nanoparticles can be synthesized by traditional methods, such as chemical and physical routes, or more recently by biogenic processes. A great variety of macro and microorganisms can be successfully used as reducing agents of metal salt precursors in the biogenic synthesis of metal-based NPs for antimicrobial activity. Depending on the nature of the biological agent, NPs with different sizes, aggregation states, morphology, surface coatings and charges can be obtained, leading to different antimicrobial effects. Considering the drug resistance to traditional therapies, the development of versatile nanomaterials with potent antimicrobial effects is under intensive investigation. In this sense, this review presents and discusses the recent progress in the preparation and application of metal-based nanoparticles biogenically synthesized for antibacterial and antivirus applications. The strength and limitations are critically discussed.

## 1. Introduction

In the last decades, engineered nanoparticles (NPs) have been extensively used in different fields such as electronic, medicine, pharmaceutical, agriculture, textiles, and cosmetics. Among them, metal-based nanoparticles such as silver (AgNPs), copper or copper oxides (CuNPs or CuO-NPs), iron oxide (FeO-NPs), gold (AuNPs), zinc oxide (ZnO-NPs), and titanium oxide (TiO_2_-NPs) have received particular attention due to their impact in human health including several applications as antimicrobial agents [1,2]. These NPs can be prepared by different methods which impact the nanoparticle features and biological responses. Mainly, metal-based nanoparticles have been synthesized using chemical and physical routes [3,4,5,6,7,8,9,10]. These methods can be harmful to human health and to the environment, restricting their use in biomedical applications since that is where they are usually employed [11]. In a similar fashion, physical routes demand a high energy input.

**Figure 1:**
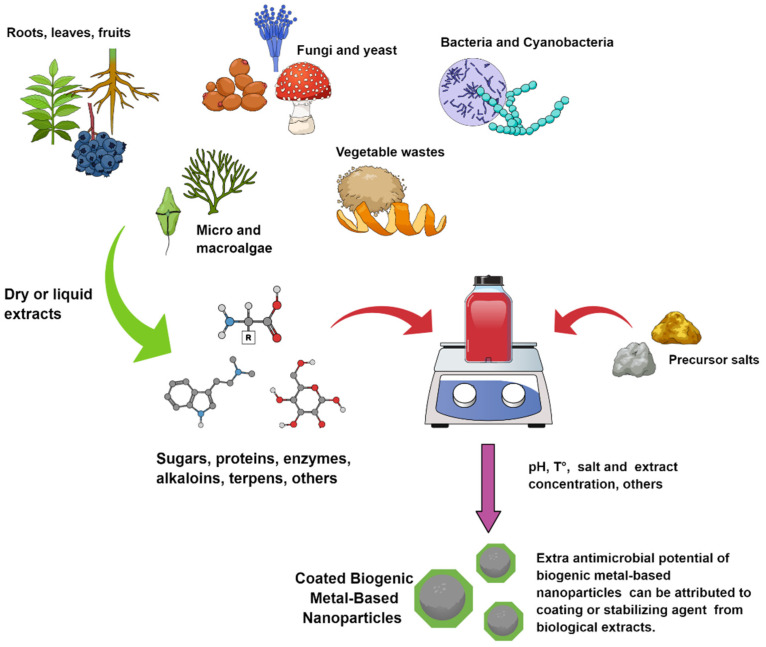
Main biological sources used to prepare biogenic metal-based NPs.

In contrast, eco-friendly alternatives have attracted the attention of the scientific community. In this direction, biogenic (or biological) methods are considered cost effective and non-toxic due to their simplicity and the absence of toxic reagents. In this regard, a plethora of biological resources have been evaluated in the biogenic synthesis of metal-based NPs (Figure 1) such as, plants, bacteria, yeast, fungi, macro and microalgae and waste materials [12,13,14,15,16].

In a typical biogenic route, the biological entity acts not only as a reducing agent (yielding to the NPs), but also as a capping agent, coating the NPs surface, avoiding their oxidation, degradation and enhancing their biocompatibility [17,18]. In addition, depending on the nature of the biological entity used, an extra feature can be given to the NP´s surface, such as an improvement of their antimicrobial activity, compared to the NPs synthesized by chemical or physical routes. For example, an improved antibacterial activity with lipoic acid capped AgNPs against *Staphylococcus epidermidis* and *Streptococcus mutans*, compared with AgNPs coated with polyethylene glycol or tannic was reported [19]. These biogenic synthesized NPs can find important biomedical and agricultural applications in the control of diseases caused by bacteria or viruses [2,20]. Indeed, in some cases, metal-based NPs, synthesized by biogenic routes, demonstrated higher antimicrobial activity related to the same metal-based NPs synthesized by the chemical route [21]. In the biogenic synthesis of nanomaterials, the biological entity acts not only as the reducing agent yielding the nanoparticles, but also as the capping agent of the obtained NP, avoiding NP aggregation and agglomeration. Moreover, depending on the chemical nature of the biological entity, an additional feature can be provided for the final nanomaterial, such as antioxidant and anti-inflammatory actions [22].

**Figure 2:**
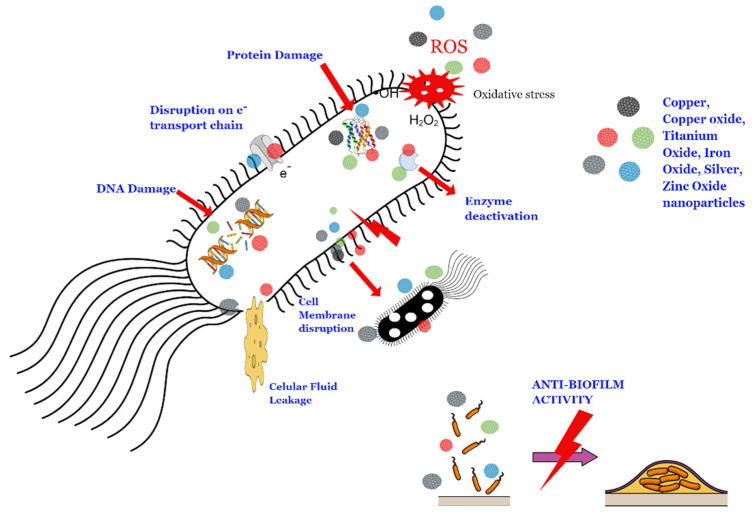
Schematic representation of the damage to bacterial cells caused by metal-based NPs.

Biogenic metal-based NPs have shown to be efficient compared with common antiseptic agents used in medicine [23]. For instance, biogenic AgNPs incorporated into an ointment base have been used for the promotion of wound healing in the acceleration and suppression of wound infections. The formulation showed a quickly wound closure rate, as well as an efficient antiseptic in the wound infection bed against several pathogenic bacteria [24]. Biocompatibility assays in rats have shown that biogenic NPs did not represent a significant toxicity in terms of hematological and biochemical parameters, as well as an excellent biocompatibility with human red blood cells [25]. The antimicrobial mechanisms of action of metal-based NPs have been associated with the death of pathogens through membrane disintegration and the generation of reactive oxygen species (ROS) leading to cell death [26,27], as represented in Figure 2. In addition, the inhibition of bacterial kinase by biogenic ZnO NPs has been recently reported as a possible antimicrobial mechanism of action [28].

**Figure 3:**
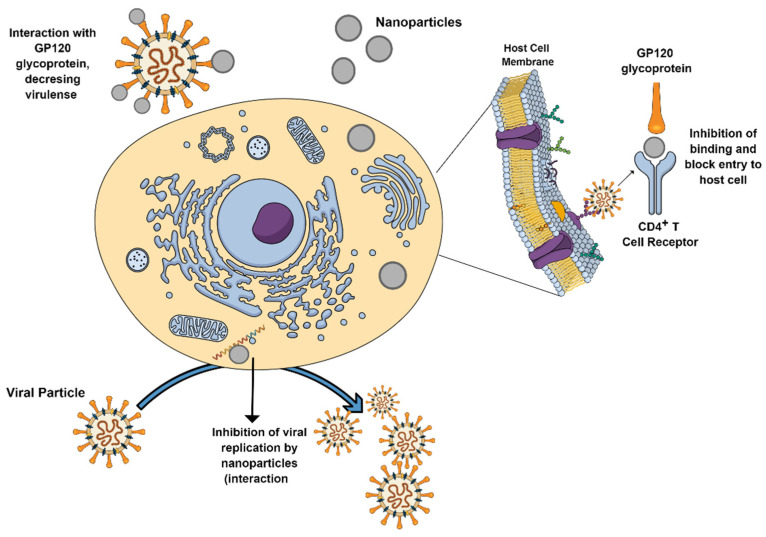
Main described action mechanisms by which metal-based NPs can act against viruses.

The antiviral capacity of metal-based NPs has also been reported [2,29]. Biogenic nanoparticles have proved to be efficient against the Herpes Simplex virus (HSV-1), the Newcastle virus (NVD), the Coxsackievirus B4 virus, the hepatitis A virus, and the chikungunya virus, among others [17,30,31,32,33]. The proposed action mechanisms by which metal-based NPs can act against viruses are shown in Figure 3. Biogenic NPs also have a potent antimicrobial activity against plant pathogens. For instance, fruit extract AgNPs demonstrated excellent antimicrobial activities against *Bacillus cereus* and *Pseudomonas syringae*, as well as against *Listeria monocytogenes* and *Staphylococcus aureus*, for which they have been proposed as an excellent additive in food packaging materials [34,35]. Viral diseases in plants have been also reported [2]. In this regard, ZnO-NPs biogenically synthesized using *Mentha spicata* extract have proved to be an efficient treatment against the Tobacco Mosaic Virus (TMV) with a 90.21% reduction in the level of viral accumulation and disease severity in treated plants, compared with untreated ones [36].

In this scenario, this review presents and discusses the current knowledge and advances in the last five years in the field of the antibacterial and antiviral activities of biogenic synthesized metal-based NPs. Moreover, new insights into the pathogenic resistance to biogenic NPs are also discussed.

## 2. Biogenic Synthesis of Metal-Based NPs

The biogenic synthesis of metal-based NPs has proved to be an innovative technology, biologically safe, biocompatible with the environment, and cost-effective compared to chemical and physical methods [37]. This method reduces the time of synthetic procedure, the costs, and provides the direct NP surface functionalization with the biological entities involved in the synthesis, which usually consists of an additional procedure in the physical or chemical synthesis [38,39].

Plant, bacteria, fungi, yeast, virus, and algae are the principal biological resources to biogenically synthesize metal-based NPs (Figure 1) [40]. The biogenic synthesis is based on metabolic processes carried out in plants and microorganisms to resist or adapt to the high concentration of toxic metals found in the environment [37,41]. Studies have reported the efficient use of reducing and stabilizing agents of microorganisms and plants by biological mass or extract for producing biogenic NPs through a sustainable technique [41]. The synthesis is carried out through the redox processes of metallic ions by secreted molecules such as sugar, carbohydrates, proteins, among others, but the full mechanism is not understood so far [40]. For instance, plant extracts are rich in polyphenols that act as strong reducing agents of metal ions leading to the formation of metallic NPs [42]. In addition, the reduction of metallic ions can be produced by cell components as amine, carbonyl, phenolic, pigments, terpenoids, alkaloids, among others. Nevertheless, the great chemical variety of metabolites involved in the reduction process makes it difficult to elucidate the mechanism of action [38]. Moreover, the experimental conditions (i.e., pH, temperature, reactant concentration, and reaction time) play an important role in the physical–chemical properties of the obtained NP [37].

As there are a great variety of biological entities needed to synthesize metal-based NPs, the ability and facility to easily scale up the production should be relevant in the choice of the best biogenic route [20]. In this sense, the use of plants as a resource to synthesize NPs offers important advantages compared to other biological entities, such as the low-cost effects, the ability to scale up the synthesis, and the presence of a greater number of active metabolites to the reduction process [43]. Furthermore, the biodiversity and the availability of different plant families with different profiles of primary and secondary metabolites contribute to the production of a greater variety of biogenic NPs with important antimicrobial applications [44]. Plant crude extracts are important sources of secondary metabolites such as flavonoids, terpenoids, alkaloids, phenol, steroids, saponins, among others, which can have anti-inflammatory, antioxidant, and antimicrobial activities [20]. Furthermore, plant extracts can be obtained from different parts of the plants, such as the seeds, stems, leaves, roots, shoots, flowers, and fruits. Consequently, the physicochemical properties of the obtained NPs nanoparticles depend on the species and the part of the plant used for the biosynthesis, which influence the NP shape, size, and surface. Diverse studies have reported the use of plant extracts to synthesize AgNPs, CuNPs, CuO-NPs, FeO-NPs, AuNPs, ZnO-NPs, and TiO_2_-NPs, among others [11]. For instance, biogenic AgNPs and AuNPs were synthesized using plant extracts (Singh et al., 2018ab). Indeed, the plant species *Acalypha indica*, *Eucalyptus citriodora*, *Garcinia mangostana*, *Morus*, and *Tanacetum vulgare* were used to synthesize AgNPs with antimicrobial activity [26,45]. In a similar manner, the antimicrobial activity of ZnO NPs synthesized by plant extracts from *Azadirachta indica*, *Trifolium pretense*, *Padina tetrastromatica*, and *Solanum nigrum*, among others, has been reported [46]. In the same way, TiO_2_ NPs synthetized with *Azadirachta indica* leaf extract have shown important antimicrobial activity [47].

In addition, microorganisms can biosynthesize NPs due to their reductase enzymes, which detoxify accumulated heavy metals [48]. Indeed, the microbial synthesis of NPs by secondary metabolites is performed at room temperature and pressure as part of an environmentally friendly and economic cost-effective approach [49]. Moreover, environmental conditions, such as stress conditions can trigger microorganisms, such as bacteria, to enhance their production of extracellular molecules aimed at their survival. These molecules are of particular interest for the generation of NPs. Microbial reductase enzymes can reduce metal salts to metal, and in most of the cases, this process can lead to NPs with a narrow size distribution. Overall, microorganisms can produce metal-based NPs through intracellular or extracellular routes [50]. The intracellular pathway is carried out by the ion transportation into the microbial cell for the metal ions to reduce to their elemental form by enzymes and electrostatic interactions. Meanwhile, the extracellular pathway involves the enzymatic reduction, which occurs on the cell surface or by the excretion of molecules able to reduce metal ions [50,51]. In a typical procedure, the extracellular synthesis is carried out by mixing the microbial culture filtrate and aqueous solution of the metallic salt. Meanwhile, intracellular synthesis involves the washing of microbial biomass and its incubation with a metal ion solution [41]. A variety of bacteria, yeast, and fungi have the ability to produce biogenic NPs with antimicrobial activity, such as *Pseudomonas stutzeri*, *Bacillus subtilis*, *Escherichia coli*, *Aspergillus flavus*, *Fusarium oxysporum*, *Verticillium sp.*, and *Schizosaccharomyces pombe*, among others [52]. Furthermore, the live and dead biomasses of algae species have become increasingly relevant in the biogenic synthesis of NPs in the last few years. The algae species are characterized by having a good metal uptake [53]. The biosynthesis of NPs by the aqueous extract of algae is mediated by metabolites such as steroids, tannins, proteins, and polysaccharide, among others [53,54]. Factors such as their easy cultivation, fast growth, and the great possibility of production scaling have attracted attention in the biosynthesis of NPs mediated by algae species [55]. Interestingly, the screening of microalgae species for the biosynthesis of Ag/AgCl nano hybrids with antibacterial effects has been reported [56]. The results indicated that *Chlorella* sp. was an effective resource for the biogenic synthesis of Ag/AgCl nano hybrids with antibacterial activity in *Bacillus* species and *E. coli* [56].

Table 1 summarizes some relevant and recent research studies based on the biogenic synthesis of metal-based NPs using plant extracts (leaf, roots, and fruits), fungi, bacteria, algae, and oomycetes, among others. As stated before, the size distribution of metal-based NPs strongly depends on the bio-compounds present in the extract. The presence of a strong or weak reductant agent in the extract can promote a fast or slow reaction rate, favoring the formation of smaller or bigger NPs, respectively [57]. In this sense, the different optical bandgaps, energies, and morphologies of biogenic CuO-NPs depend on the nature of the vegetable extract waste used for the NP biosynthesis [58].

As described above, there are a large number of biological resources for the synthesis of NPs and their physical–chemical properties depend on many factors. Consequently, analytical techniques should be applied for the characterization of NPs [86]. In general, some techniques such as UV–Vis spectroscopy, X-ray diffraction, Fourier transform infrared, energy dispersive X-ray spectroscopy, scanning electron microscopy, transmission electron microscopy, and dynamic light scattering have been widely used by researchers [87] (Table 1). For example, UV–Vis spectroscopy allows the optical properties to be determined, while X-ray diffraction provides information about the crystalline structure of the NPs and, consequently, the chemical composition of the obtained NP [88]. Otherwise, energy dispersive X-ray spectroscopy examines the elemental composition, and Fourier transform infrared provides information about the surface composition and ligand binding, which is an important technique not only regarding the chemical properties of the NPs, but also for investigating the capping composition. In addition, microscopic techniques provide morphological characterizations such as shape, size, aggregation state, and surface properties. Finally, the dynamic light scattering technique is widely used to determine the hydrodynamic size, homogeneity, surface charge, and stability over time of NPs in suspension, allowing the characterization under different conditions of temperature and pH, among others to be determined [89].

## 3. Bactericidal and Virucidal Activities of Biogenic Metal-Based NPs

The antimicrobial actions of metal-based NPs are based on the release of metal ions, cell membrane damage, DNA interaction/damage, and free radical generation. Further discussions on the mechanisms of the toxicity of NPs can be found elsewhere [2,27]. This section discusses selected examples of recent papers based on the antimicrobial activity of different metal-based NPs synthesized by biogenic routes.

### 3.1. Bactericidal and Virucidal Activities of AgNPs

Biogenic AgNPs are interesting eco-friendly tools that can combat pathogenic bacteria and viruses, including multi drug-resistant bacteria, which have appeared due to the overuse of antibiotics against infectious diseases, or the overuse of pesticides against pest plants. AgNPs synthesized using cyanobacterium *Leptolyngbya* sp. WUC 59 cell-free extract suppressed the growth of *Bacillus subtilis* and *Escherichia coli* bacteria, at 10 mg L^−1^ and with a minimum inhibitory concentration (MIC) of 8 mg L^−1^ [39]. Antibacterial assays with AgNPs synthesized by using *Calligonum comosum* roots and *Azadirachta indica* leaf extracts were effective against *Pseudomonas aeruginosa*, *Escherichia coli*, and *Staphylococcus aureus* at concentrations that ranged between 10.9 and 21.4 mg L^−1^ [61]. A double-green approach for the biosynthesis of AgNPs has been proposed by the authors of [90]. AgNPs were biosynthesized using chitosan as a reducing agent, which was obtained from the carapace of marine crabs *Portunus pelagicus* and combined with the microwave technique. The AgNPs obtained ranged between 7 and 25 nm in size, with near-spherical shapes, and showed an efficient antimicrobial activity against *Edwardsiella tarda* and *Escherichia coli*. AgNPs derived from *Albizia lebbeck* Bark extract (average size of 27 nm) showed antimicrobial activity against multi-drug resistant bacteria *P. aeruginosa* and *S. marcescens*. The proposed antibacterial activity was assigned to the efficient penetrability of the NPs on the bacterial cell membrane. Indeed, the cell damage was confirmed by demonstrating the significant alterations in the cellular architecture, as assayed by scanning electron microscopy [91]. In a similar work, AgNPs (30 mg) synthesized by using *Allium sativum* clove extract were effective against *Klebsiella pneumoniae*, *Pseudomonas aeruginosa*, *Serratia marcescens*, *Streptococcus pyogenes*, and *Staphylococcus aureus* [62]. Interestingly, this work not only demonstrated the antimicrobial capacity of freshly prepared AgNPs, but also demonstrated that the antimicrobial capacity of NPs was maintained after AgNPs were stored at 4 °C, 22 °C, and 37 °C or even at thermal treatment at 300 °C, 500 °C, for 1 h.

The difficulties related to the treatment of bacterial infection in the urinary tract due to the inaccessibility of traditional antibiotics to reach urinary tract infections caused by the uropathogenic *Escherichia coli*, which causes biofilm formation, were efficiently overcome by using AgNPs biogenically synthesized with a fungal extract from *Fusarium scirpi* [60]. AgNPs showed a MIC of 25 mg mL^−1^ over planktonic bacteria, but a sub-MIC concentration of only 7.5 mg L^−1^ was needed to cause a 97% inhibition in the formation of the biofilm or to produce 80% of the mature bacterial biofilm. An innovative strategy against biofilm-forming multidrug resistant bacteria was proposed by the authors of [92]. The authors biosynthesized small AgNPs (13.47 ± 12 nm) using the supernatants of *Bacillus horikoshii* AJM-A1 and conjugated them with the alpha-amylase enzyme from *Bacillus subtilis*. Conjugated AgNPs were evaluated on multidrug-resistant strains *Klebsiella pneumoniae* and methicillin-resistant *Staphylococcus aureus* (MRSA). The results demonstrated that conjugated AgNPs at concentrations that ranged between 200 μg mL^−1^ and 800 μg/mL^−1^ caused a biofilm reduction between 80 and 95%, approximately, and were more effective than non-conjugated AgNPs or alpha-amylase independently [92]. Similarly, AgNPs (48–67 nm diameters) synthesized from leaf extracts of *Catharanthus roseus* and *Azadirachta indica* inhibited the growth of *E. coli*, *K. pneumoniae*, *S. aureus*, and *P. aeruginosa* isolated from patients with septic wound infections [93]. However, *P. aeruginosa* was inhibited by higher concentrations of AgNPs (80 µg µL^−1^), compared to *E. coli*, *K. pneumoniae* and *S. aureus* that were inhibited at 10 µg µL^−1^ of AgNPs.

Interestingly, AgNPs can also act as agents to recover the antimicrobial activity of antibiotics on resistant bacteria. In this sense, AgNPs synthesized by using a *Rosa damascenes* extract and conjugated with cefotaxime were efficient against cefotaxime-resistant *E. coli* strains. Conjugated AgNPs (8.48–25.3 nm) showed a higher antimicrobial activity against resistant bacteria, compared to non-conjugated AgNPs or pure antibiotics, restoring the efficiency of the cefotaxime [94].

Moreover, AgNPs have also been loaded onto graphene oxide (GO) nanomaterials. AgNPs synthesized by a coffee extract (average size of 70 nm) were incorporated on GO sheets. The nanocomposite showed bactericidal activity on *Staphylococcus aureus* due to the release silver ions, as well as photobactericidal effects due to photocatalytic activity induced by the blue light-irradiation [95]. Since AgNPs and regenerated silk fibroin have shown potential applications in preventing wound related infections, a photo-assisted green synthesis of silver doped silk fibroin/carboxymethyl cellulose nanocomposite hydrogels has been reported [96]. Silk fibroin was obtained from raw Bombyx mori cocoons, and tyrosine residues on silk fibroin served as the reductant agent of silver ions, which were exposed to UV radiation to complete the green synthesis of AgNPs. The nanoparticles in the composite hydrogels presented a diameter mean value of 92 nm and demonstrated a strong and consistent antimicrobial activity against *E. coli*, *S. aureus S. epidermidis*, *MRSA*, and *P. aeruginosa.* The authors suggested that this composite can potentially be used as a wound dressing with antimicrobial and regenerative properties [96]. However, further studies are required.

Recent important works have also shown thev antimicrobial activity of biogenic synthesized AgNPs on pathogenic plant bacteria. In this regard, AgNPs (12 nm) biosynthesized by the mycelial aqueous extract of agriculturally beneficial fungi *Pythium oligandrum* were effective against *Clavibacter michiganensis* subsp. *michiganensis* (Cmm) known for producing bacterial canker in tomatoes [59]. In a similar strategy, AgNPs (8–28 nm in size) were biosynthesized by using *Moringa oleifera* leaves, which act as the main reducing and stabilizing agent [97]. The obtained NPs were applied on *Citrus reticulata* inoculated with *Xanthomonas axonopodis* pv. Citri. The results demonstrated that 30 ppm of AgNPs was a suitable concentration for creating a resistance against canker disease, produced by *X. axonopodis* pv. Citri [97]. Moreover, biogenic AgNPs (25 to 50 nm in size) synthesized by using the endophytic bacterium *Bacillus siamensis* strain C1 effectively inhibited the rice pathogenic bacteria *Xanthomonas oryzae* pv. oryzae strain LND0005 and *Acidovorax oryzae* strain RS, at an NP concentration of 20 mg mL^−1^ [98].

Although some progress has been achieved by from using biogenic synthesized AgNPs as antibacterial agents, the antiviral activity of these NPs has been studied less compared with the AgNPs that have been synthesized by traditional routes (chemical and/or physical methods). However, some important works have described the ability of biogenic AgNPs against pathogenic viruses. For instance, blue-green algae *Oscillatoria* sp produced spherical AgNPs (15–50 nm in size) with toxic effects against the Herpes Simplex (HSV-1) virus, causing a 90% reduction in its cytopathic effect [30]. In a similar approach, AgNPs produced by the seaweed *Sargassum wightii* algae demonstrated antiviral activity against the Herpes Simplex Virus (HSV-1 and HSV-2) [99].

Interestingly, AgNPs (5–15 nm in size) obtained from an aqueous extract from *Panax ginseng* roots assisted by the ultra-sonication method were effective against the influenza A virus (strain A/PR/8) (Sreekanth et al., 2018). A Red Sea Sponge (Amphimedon) was employed in the biosynthesis of AgNPs (8–14 nm in size), assisted by silico modeling and metabolic profiling [100]. The NPs displayed protease and helicase inhibition activity against the hepatitis C virus (HCV), due to the presence of bio-compounds with anti-HCV activity in Amphimedon [94]. Other viruses such as the chikungunya virus, white spot syndrome virus, and dengue virus have also been effectively treated with biogenic AgNPs [17,101,102]. Further studies are required on this topic.

### 3.2. Bactericidal and Virucidal Activities of CuNPs and Copper Oxides CuO-NPs

Spherical CuNPs (2–10 nm) were prepared by using an aqueous extract from *Vaccinium myrtillus* L. (bilberry) and *Vaccinium uliginosum* L. subsp. *Gaultherioides* (false bilberry) and demonstrated high and broad antimicrobial activity against both Gram-negative and Gram-positive bacteria. Biosynthesized CuNPs reduced colony-forming units (CFU) mL^−1^ >3 in *S. aureus*, *E. coli* and *S. cerevisiae*, after 1 to 3 h of incubation [103]. CuNPs synthesized using a *Manilkara zapota* leaf extract resulted in spherical NPs with an average size of between 19 and 42 nm, and exhibited antibacterial activity against *E. coli*, *B. subtilis*, *S. aureus*, *V. harveyi*, and *V. parahaemolyticus* bacterial strains [104]. In a further work, CuNPs were synthesized by using a *Hagenia abyssinica* (Brace) *JF. Gmel*. leaf extract. This process led to the formation of a mixture of spherical, hexagonal, triangular, cylindrical, and irregular CuNPs with an average particle size of 35 nm. These NPs (at 1 mg mL^−1^) showed antimicrobial activity on *S. aureus*, *Bacillus subtilis*, *E. coli*, and *P. aeruginosa* [70]. The leaf extract of *Syzygium cumini* favors the synthesis of spherical CuNPs (28−35 nm) and was shown to form excellent antimicrobial compounds against methicillin- and vancomycin-resistance *S. aureus* [105]. The *Cissus vitiginea* leaf extract proved to be a suitable strategy for the synthesis of spherical shape CuNPs (5–20 nm in size). The NPs were efficient as antimicrobial agents against several urinary tract infection pathogens such as *E. coli*, *Enterococcus sp.*, *Proteus sp.*, and *Klebsiella sp* [69]. In an interesting recent work, CuNPs were generated in situ on hybrid cellulose nanocomposite films and loaded with tamarind nut powder as reinforcing fillers [106]. The use of pectin from citrus peel as a stabilizer agent, citric acid as a reducing agent, and microwave irradiation were proposed as a green and efficient tool in the biosynthesis of CuNPs [107]. Synthesized NPs (40.9  ±  13.6 nm) with irregular polygon shapes showed high antimicrobial activities against *E. coli* and *S. aureus.* Interestingly, plants with a historical capacity for use in cutaneous wound healing, such as *Allium eriophyllum* Boiss, have been used in the synthesis of CuNPs to evaluate their antimicrobial potential. There is also the possibility to use these NPs in cutaneous wound healing [108]. The authors reported the effective formation of small CuNPs (25–35 nm) using an *Allium eriophyllum* Boiss leaf aqueous extract, and potent antimicrobial activity (>4 mg L^−1^) against *P. aeruginosa*, *S. aureus*, *B. subtilis*, *S. pneumonia*, *E. coli*, and *S. typhimurium*. Moreover, ointment application containing CuNPs raised the wound contracture, hydroxyl proline, vessel, hexosamine, hexuronic acid, fibrocyte and fibrocytes/fibroblast rate, and significantly decreased the wound area, total cells, neutrophils, and lymphocytes, accelerating tissue repair [108]. The *Cardiospermum halicacabum* leaf extract produced CuNPs (30–40 nm in size), which demonstrated antibacterial activity against *P. aeruginosa* and *S. aureus*, with a high susceptibility at an NP concentration of 50 µg mL^−1^. Moreover, anti-biofilm capacity was also demonstrated, since 100 µg mL^−1^ of CuNPs inhibited 79%, 72%, and 78% of *P. aeruginosa*, *S. aureus*, and *E. coli* biofilm formation, respectively [109]. The incubation of CuO–NPs (15–25 nm in size at 100 μg mL^−1^) synthesized from the *Camellia japonica* plant leaf extract decreased the viability of the uropathogens *P. aeruginosa* and *K. pneumoniae*, in addition to the promotion of cell membrane damage [110]. As can be observed, most of the studies reported the antibacterial activity of biogenic CuNPs, and more studies based on biogenic CuO NPs should be further investigated.

Regarding the antiviral effects of CuNPs and CuO–NPs, more studies are welcome since most of the studies have focused on chemically synthesized NPs and scarce information in the literature is related to biogenic NPs [111]. The fruit extract of *Syzygium alternifolium* (Wt.) Walp. was used in the biosynthesis of spherical CuO–NPs (2–70 nm in size) with a negative zeta potential (−49 mV). These NPs demonstrated antiviral activity against the Newcastle Disease Virus (NDV). *In ovo* assays showed that an egg treated with CuO–NPs (at 25 µg mL^−1^) led to embryo deaths at 24, 48, and 96 h of incubation [73].

### 3.3. Bactericidal and Virucidal Activities of ZnO-NPs

ZnO–NPs have attracted special attention due to their low cost, photochemical stability, and photocatalytic activity. This last property has also conferred to ZnO–NPs antimicrobial capacities due to the possibility to generate reactive oxygen species (ROS), such as superoxide anion (•O_2_^−^), hydroxyl radical (•OH), and hydrogen peroxide (H_2_O_2_), upon NP exposition to UV irradiation [112]. Moreover, ZnO–NPs can produce antimicrobial activity by their direct interaction with microorganisms upon the release of Zn^2+^ ions, without the need for UV light exposure [113]. Interesting, the photocatalytic performance of ZnO nanostructures under visible light irradiation can be promoted and enhanced by doping these NPs with transition and noble metals [106]. Detailed information about several synthetic routes, photocatalytic activity, and details about the most used dopants (metal and non-metals) were extensively reviewed by the authors of [112].

*Eucalyptus globulus* essential oil was used to synthesize ZnO–NPs (24 nm), which showed efficient antibacterial activity at a concentration of 100 μg mL^−1^ against *K. pneumoniae* [114]. NPs showed an important anti-biofilm formation with biofilm inhibition of 85% and 97% against *S. aureus* ATCC 25923 and *P. aeruginosa* ATCC 27853, respectively. Similar results were obtained with hexagonal ZnO–NPs (50–100 nm) synthesized using the alcohol-free *Artemisia pallens* plant extract as a reducing agent [115]. The zone inhibition method was carried out to evaluate the antimicrobial activity demonstrating that the zone inhibition against *B. subtilis*, *S. aureus*, and *E. coli* was 12 mm, 7 mm, and 6 mm, respectively. Moreover, the determined MIC values were 31.25 µg mL^−1^ for *S. aureus* and 62.5 µg mL^−1^ for *B. subtilis* and *E. coli*. Multi-drug-resistant bacteria isolated from patients in nursing care such as *Acinetobacter baumannii* was efficiently treated with ZnO–NPs (18 nm) synthetized by the *Caryophyllus aromaticus* leaf extract and showed a MIC value of 0.19 µg mL^−1^ [116]. Moreover, anti-biofilm activity was assessed against the multi-drug-resistant *A. baumannii* as well as five clinical strains of *A. baumannii* ATCC 19606. The results demonstrated that only 1/8 of the MIC value (0.012 μg mL^−1^) of ZnO–NPs was efficient to reduce the biofilm production. Moreover, at 1/2 of the MIC (0.045 μg mL^−1^) and 1/4 of the MIC (0.023 μg mL^−1^) biofilm formation was considerably reduced in the six-strains evaluated [116]. In other works, ZnO–NPs (15–20 nm), synthesized using the leaf extract of *Coleus amboinicus*, demonstrated effective antimicrobial activity against *Salmonella typhi* (B-4420), *Klebsiella pneumoniae* (ATCC-27738), *Escherichia coli* (O157: H7), *Staphylococcus aureus* (6538 P), and *Bacillus cereus* (ATCC 7064). In addition, these NPs were used for the antimicrobial treatment of burn wound infections, accelerating wound closure rate [117].

Nanoflowers are newly developed 3-D nanostructures that have shown high stability and important biomedical applications [118]. In this regard, the biosynthesis of ZnO nanoflowers was effective by using the *Withania coagulans* extract as a reducing agent [119]. Nanoparticles with a size range of 360–550 nm and with a Wurtzite hexagonal structure were formed. Antimicrobial activity was obtained with 5, 10, and 20 μg mL^−1^ of ZnO nanoflowers against *S. aureus* and *P. aeruginosa*, which was attributed to the induced oxidative stress produced by ZnO nanoflowers and by the enhanced surface area to volume ration of the protruding projections as asteroideal nano-petals.

As a strategy to enhance the antimicrobial activity of ZnO–NPs, these NPs have been doped with several compounds, forming nanocomposites, nanoneedles, and hybrid NPs, among others. However, this strategy has been applied only with ZnO–NPs synthesized by chemical routes (Li et al. 2020b) and should be extended to biogenic synthesized NPs [112]. Interestingly, the preparation of nanoneedles composed by chitosan and Zn doped with bismuth oxide (CS/Zn_0.75_Bi_2_O_3_ nanoneedle) has been reported [120]. The nanoneedle showed inhibitory effects against *S. aureus* and *E. coli* with inhibition zones of 32 and 35 mm, respectively. Sericin, a natural protein produced by the silkworm, *Bombyx mori*, was used to biosynthesize Ag/ZnO hybrid NPs on a sericin/agarose composite film [121]. The composite was characterized by its high hydrophilicity and water absorption ability, as well as its favorable mechanical properties. Moreover, the composite showed strong antimicrobial activity against *S. aureus* and *E. coli*, where the disruption of the bacterial cell wall integrity was observed, due to the synergistic effect of AgNPs and ZnO–NPs [121]. Recently, the preparation of curcumin-loaded ZnO–NPs (73 nm in size and 654 m^2^ g^−1^) decorated with mesoporous silica as a tissue adhesive, has been reported [116]. The results found in animal studies showed a significant antimicrobial activity of the nanomaterial as well as its effectivity for gluing wounds in less than one minute and healing within five days [122]. Zinc oxide-xanthan gum nanocomposite, formed by a green route, at a concentration of 128 µg mL^−1^ showed a high capacity to reduce quorum sensing related to virulence factors, such as chitinase 70% and violacein (61%) in *Chromobacterium violaceum* and protease (72%), and prodigiosin (71%) in *Serratia marcescens* [123].

ZnO–NPs have also been applied to control pathogenic plant bacteria. In this sense, ZnO–NPs synthesized by using the *Thymbra spicata* var. spicata L. (TS) plant extract showed a hexagonal nanorod-like structure between 426 nm and 540 nm in diameter and antimicrobial activity against several plant pathogenic bacteria such as *P. syringae* pv. *Phaseolicola* (PspE22), *P. carotovorum* subsp. *Carotovorum* (PccSy17), *C. michiganensis* subsp. *Michiganensis* (CmmAd12), and *P. cichori* (PcSa2) [124]. Interestingly, the authors reported that pristine ZnO–NPs were less effective than biosynthesized ZnO–NPs. Finally, many works have reported virucidal activity by ZnO–NPs. Indeed, the antiviral capacity of ZnO–NPs against HSV-1 and HSV-2, human rhinovirus (HRV), and human immunodeficiency viruses (HIV), has been already reviewed [125]. However, all the works were developed with chemically synthesized NPs. To the best of our knowledge, biogenic synthesized ZnO–NPs have not been tested against pathogenic viruses. Considering the advantages of biogenic routes and the reported antibacterial actions of these NPs, further studies that aim to treat human and plant viruses with biogenic synthesized ZnO–NPs are welcome.

### 3.4. Bactericidal and Virucidal Activities of TiO_2_–NPs

The antimicrobial effect of biogenic TiO_2_–NPs synthesized with an extract of Vitex negundo Linn with or without the addition of ionic liquid (1-ethyl-3-methylimidazolium tetrafluoroborate-[EMIM]+BF4-) against *S. aureus* and *E. coli* has been reported [126]. The ionic liquid was designed to act as a self-assembling template agent during the synthesis of TiO_2_–NPs. Microscopy revealed that both NPs presented a rod-shape and spherical morphologies (size in the range of 15–26 nm). The results demonstrated an important growth inhibition of both *S. aureus* and *E. coli* as assayed by agar diffusion assays [126]. Similarly, the leaf extract of *Morinda citrifolia* was used to prepare quasi-spherical TiO_2_–NPs (15–19 nm in size), which demonstrated antibacterial activity at an NP concentration from 50 to 150 μg mL^−1^ against *S. aureus*, *E. coli*, *B. subtilis*, and *P. aeruginosa*, in a dose–response manner [127]. In addition, spherically-shaped TiO_2_–NPs synthesized with the aqueous leaf extract of *Trigonella foenum-graecum* showed an important activity against *E. faecalis*, *S. aureus*, *S. faecalis*, *B. subtilis*, *Yersinia enterocolitica*, *E. coli*, *P. aeruginosa*, *K. pneumoniae*, and *C. albicans* [128]. Furthermore, triangular TiO_2_–NPs (20–50 nm in size) synthesized with orange peel extract have antimicrobial activity against *S. aureus*, *E. coli*, and *P. aeruginosa* at doses from 6.75 to 50 mg mL^−1^ [129]. In addition, spherical TiO_2_–NPs (average size of 69 nm) synthesized from the root extract of *Glycyrrhiza glabra*, from 0.62 to 5 µg mL^−1^, have a good toxic effect against *K. pneumoniae* and *S. aureus* [130]. TiO_2_–NPs (spherical shape and size from 15 to 50 nm) produced by the *Azadirachta indica* leaf extract had a strong antibacterial activity against *E. coli*, *B. subtilis*, *Salmonella typhi*, and *K. pneumonia*, where the antimicrobial effect of the NP was higher compared to bulk TiO_2_. The results indicated that the lowest MIC value was 10.42 μg mL^−1^ for the NPs against S. typhi and E. coli, while the lowest MBC value was 83.3 μg mL^−1^ for the NPs against K. pneumonia. Recently, an extract from the *Mentha arvensis* leaves was used to synthesize spherical TiO_2_–NPs (20–70 nm), which demonstrated antibacterial effects against *Proteus vulgaris* at doses from 10 to 30 mg mL^−1^, in a dose–response manner. Interesting, the same NPs at the same dose had no effect towards *E. coli* or *S. aureus* [131]. These results indicate that the antimicrobial activity of biogenic TiO_2_–NPs may vary based on the bacteria strain. Moreover, TiO_2_–NPs (50–90 nm in size with spherical and square shapes), synthesized from the root extract of *Withania somnifera* demonstrated antibacterial effects against *E. coli*, *P. aeruginosa*, *S. aureus*, *Listeria monocytogenes*, and *Serratia marcescens* through the inhibition of biofilms and enhanced the formation of ROS [132].

Taken together, important papers have described the successful preparation of biogenic TiO_2_–NPs, mainly by a plant-mediated synthesis, leading to the formation of particles at the nanoscale with different morphologies and potent antimicrobial activity. However, biogenic synthesized TiO_2_–NPs have not been used as antiviral agents, and studies on this topic are welcome.

### 3.5. Bactericidal and Virucidal Activities of FeO-NPs

Iron-based nanoparticles (FeO–NPs), such as magnetite (Fe_3_O_4_), hematite (Fe_2_O_3_), and maghemite (γ-Fe_2_O_3_) NPs have been extensively used in different areas including biomedical applications due to their superparamagnetic properties at room temperature [133]. These NPs are different phases of iron oxide with similar crystallographic structures [134]. Recently, in addition to chemical and physical routes, biogenic methods have been used to prepare superparamagnetic FeO–NPs [135,136]. The main biomedical applications of FeO–NPs are in cancer diagnoses and treatment, however, more recently these NPs have been explored as antimicrobial agents, as described below.

α-Fe_2_O_3_–NPs (average size of 16 nm) with asymmetric morphology and uniform dispersion, were obtained from the *Sida cordifolia* plant extract, which is rich in carbohydrates, alkaloids, proteins, glycosides, tannins, terpenoid, and flavonoids [137]. The proposed mechanism of NP formation involves the formation of an octahedral aqua complex of Fe (III), which undergoes a decomposition into Fe(OH)^2+^ that can form a complex with functional groups of phytochemicals derived from the plant extract. By increasing the temperature, NPs of α-Fe_2_O_3_ are formed from Fe(OH)^2+^ species. The antibacterial activity of α-Fe_2_O_3_–NPs (at 50 μg mL^−1^) was evaluated against different Gram-positive and Gram-negative bacteria (*B. subtilis*, *S. aureus*, *E. coli*, and *K. pneumonia*), and compared with traditional antibiotics (at 50 μg mL^−1^). It was observed that α-Fe_2_O_3_–NPs were efficient in the combating of Gram-positive bacteria such as *B. subtilis*, demonstrating the good antibacterial action of α-Fe_2_O_3_–NPs compared to the commercial antibiotic neomycin. Moreover, the authors have explained that these NPs are stable in the environment and have a lesser contribution in the release of metal ions for antibacterial action. However, UV light activates reactive oxygen species, leading to the desorption of the bacterial membrane, resulting in death [137]. Interestingly, the flower extract from *Nyctanthes arbor tristis*, popularly known as the “Sad Tree”, has been used to synthesize Fe_2_O_3_–NPs [138]. Field emission scanning electron micrograph (FE-SEM) images demonstrated NPs with grains from 50 to 180 nm, indicating the formation of clusters with spherical morphology. The obtained NPs demonstrated antimicrobial activity against *K. pneumoniae* and *S. aureus*, at a concentration of 100 µg mL^−1^. The NPs were found to be more efficient against the Gram-positive bacteria *S. aureus* compared to the Gram-negative *K. pneumoniae* [138].

*Eucalyptus robusta* leaf extracts (in the proportion of 1:1, 2:1, and 1:2 in relation to the iron salt) were used to synthesize iron NPs (Fe–NPs) [139]. The obtained NPs have a spherical morphology with average size of 8 nm, at a solid state, along with the presence of some clusters with a width of 70 nm, leading to the formation of some degree of aggregates. These NPs synthesized under the different conditions were tested against *P. aeruginosa*, *B. subtilis*, *E. coli*, and *S. aureus*. The antibacterial action of the Fe–NPs was compared to the activity of the *Eucalyptus robusta* leaf extracts, since the phytochemicals derived from this extract are known to have antimicrobial activity. The authors demonstrated the significant antimicrobial action of Fe–NPs against the Gram-positive *B. subtilis*, indicated by the accumulation of Fe–NPs in the cytoplasmic region of the microorganism, which might lead to membrane rupture leading to the death of the microorganism. In addition, the possible mechanism of the antimicrobial activity of Fe–NPs is assigned to the generation of ROS leading to cellular oxidation and cell death. Fe–NPs demonstrated a higher antimicrobial action compared with the pure *E. robusta* extract. Moreover, the antioxidant effects of the NP were demonstrated [139].

In another study, spherical α-Fe_2_O_3_–NPs (average size of 100 nm) were obtained from *Plectranthus amboinicus* (Mint) [140]. Scanning electron microscopy showed the absence of aggregates, which was associated with the capping of the NPs with phytochemicals derived from the plant extract. The antimicrobial activity of the NPs was demonstrated against *Klebsiella*, *Salmonella Typhi*, *E. coli*, and *S. aureus* [140].

In addition to their antimicrobial effect against planktonic bacteria, FeO–NPs also have toxic effects against bacterial biofilms, including the prevention of biofilm formation [141]. Due to their superparamagnetic behavior, the application of an external magnetic field can conduct FeO–NPs allowing their permeation into bacterial biofilms [142]. This is an interesting application of superparamagnetic FeO–NPs that should be further explored.

As for the virucidal actions of Fe–ONPs, recently the anti-virus activity of FeO–NPs synthesized by chemical routes has been demonstrated [143]. New investigations based on the anti-virus actions of biogenically synthesized FeO–NPs are needed. Taken together, important recent publications have described the successful preparation of FeO–NPs by biogenic synthesis mediated by plant leaf extracts leading to materials at the nanoscale with potent antimicrobial actions against different bacteria strains. Further studies are required in relation to the anti-virus action of these NPs.

### 3.6. Other Biogenic Synthesized Metal-Based NPs (NiO-NPs, Pd-NPs and SnO_2_-NPs)

Other biogenic metal-based NPs have been explored in relation to different biomedical applications, including antimicrobial activity. For instance, the antibacterial effect of nickel oxide NPs (NiO–NPs) synthesized from the stevia leaf extract has been demonstrated against *B. subtilis*, *S. pneumonia*, and *E. coli* [144]. NiO–NPs were prepared with a broth that originated from the stevia leaf extract in the presence of nickel acetate, leading to the formation of spherical NPs with sizes between 10 and 40 nm. The authors observed a more effective antimicrobial effect of NiO–NPs against the Gram-negative *E. coli*, compared to the strains of *S. pneumoniae* and *B. subtilis* (Gram-positive). In fact, NiO–NPs showed a concentration-dependent toxicity with 70% bacteria eliminated at a concentration of 200 µg/mL [144].

Likewise, palladium nanoparticles (Pd–NPs), at sizes between 10 and 100 nm, can have antimicrobial effects due to their interaction with molecules outside or inside the cell surface [145]. In this sense, the *Garcinia pedunculata roxb* leaf extract was used to synthesize spherical Pd–NPs with an average size of 3 nm [146]. The antimicrobial activity of Pd–NPs has been demonstrated against the bacterium *Cronobacter Sakazakii* AMD 04. The MIC and MBC assays showed a significant inhibition in biofilm formation upon treatment with Pd–NPs (0.26 mM). A significant decrease in biofilm formation was found for Pd–NPs at 0.39 mM and 0.52 mM [146].

Similar to NiO–NPs and Pd–NPs, tin NPs (SnO_2_–NPs) also have antimicrobial activity. *Saraca indica* flowers were used in the synthesis of spherical SnO_2_–NPs (size from 2.2 to 18 nm), and their antimicrobial activity was revealed against *E. coli*, a Gram-negative bacterium with a thicker cell wall and a more complex protective membrane, indicating that these NPs have the ability to penetrate the outer membrane [147]. The authors assumed that ROS produced through the interaction between SnO_2_–NPs and the bacterial cell membrane allowed the metal NPs to penetrate into the cell [147].

Taken together, these few articles have described the successful preparation of other types of metallic nanoparticles, such as NiO–NPs, Pd–NPs and SnO_2_–NPs by biogenic routes (plant-mediated synthesis) giving rise to NPs with spherical morphologies, small sizes and with antimicrobial actions. Further studies are still welcome in relation to this topic to further explore the antibacterial action of these NPs. Moreover, we did not find biogenic synthesized NPs that were effective against viruses; hence, further studies in this area would produce important contributions.

## 4. Conclusions, Challenges and Perspectives

Nanobiotechnology is the combination of interdisciplinary principles that involve areas of knowledge related to chemistry, physics, biology, and material science, and for many decades it has been providing a purpose to innovative applications mainly in the fields of medicine and biomedicine. The development of metal and metal oxide NPs, including noble metals, such as silver, can be obtained by greener and friendlier routes in comparison to the most well-known chemical and physical syntheses. Green synthesis, also known as biogenic synthesis, is one of the most discussed alternatives at the moment for obtaining NPs with medical relevant applications. As stated in this work, biogenic routes are considered less toxic, cleaner, more economically viable and a scalable type of route, compared with traditional methods (chemical and physical routes). A typical biogenic route can use plant extracts, algae, yeasts, fungi, and bacteria. The biological entity acts as a reducing agent, reducing metal ions to the NP, and as a capping agent, avoiding NP aggregation and agglomeration. Overall, the biological mechanism of NP production is based on redox pathways involving the biomolecules of the biological agent and metal ions, which are the NP precursors.

Although much progress has been achieved in the domain of biogenic synthesis of metal-based NPs of medical relevance, there are still some issues to be completely overcome. Not all biological organisms can be successfully used in the biogenic preparation of NPs. For instance, not all algae or bacteria species can be exploited for the synthesis of nanomaterials, since some of them might contain toxic compounds. Moreover, since a great variety of biological entities can be used in the biogenic synthesis of NPs, the mechanism for biogenic synthesis can vary depending on the starting biological material employed, and thus it has not been fully understood. As the chemical redox ability of the biological entity can vary, the obtained nanomaterial can have different chemical and physical features, depending on the starting experimental conditions employed. Thus, a great challenge in the biological synthesis of nanomaterials is the exact control over nanoparticle size distribution, morphology, and chemical surface. In this sense, chemical and physical routes might present a superior ability to control the nanoparticle feature, compared to the biological routes. Further studies are required to better control the reproducibility of the nanomaterial feature in a biogenic synthesis. In fact, the relationship between nanoparticle properties and the experimental biological conditions in a biogenic route of NP needs to be further explored. An important aspect to be further explored in the biogenic synthesis of NPs is the complete characterization of the biological agents responsible for NP production. From the revised literature, more studies aimed at a better characterization of the redox aspects of the biogenic synthesis of NP are still necessary and a complete characterization of the biological agent used in the synthesis is still poorly described. Finally, studies focused on the exploitation of the biogenic production of NPs on an industrial scale are welcome.

Metal based NPs have an important antimicrobial application. Currently, Cu, Ag, and Fe-based NPs are widely targeted for having bactericidal characteristics and some nanoparticles in particular have virucidal activity, leading to the versatility of their applications against a range of Gram-positive and Gram-negative bacterial strains and some types of viruses. When concluding that over the years some bacteria become resistant to traditional antibiotics, and the increasing problems with multi-resistant bacteria, we face the need to overcome this issue by developing new and versatile antimicrobial agents, such as metal based NPs.

Nanomaterials have the ability to exhibit intrinsic properties such as a large surface area, a reduced size, morphology, and biocompatibility at concentrations able to promote antimicrobial activity. Thus, nanomaterials have unique properties making them suitable candidates to combat a broader spectrum of bacteria and viruses, including resistant biofilms. In this sense, biogenic synthesized metal-based NPs might find important applications in medical, cosmetic, pharmaceutical, agricultural, and food sectors.

Overall, this work has summarized the recent progress in the preparation of biogenic metal-based NPs with important antimicrobial effects. Although progress has been achieved, more research in this topic should be carried out. Most of the antimicrobial effects reported herein are based on antibacterial effects, and there is a lack of studies aimed to evaluate the antivirus effects of these NPs. In addition, a comparison of the efficacy of biogenic NPs with NPs synthesized by chemical and/or physical routes should be further evaluated. Studies focused on the biocompatibility of these NPs, their fate in vivo, including biodistribution and toxicology investigation should be studied. Finally, studies to evaluate the mechanisms of action of these NPs are in high demand. Overall, it has been assumed that the small size of the NPs is an important factor that determines their biological activity towards bacteria, viruses, and proteins [148]. We hope that this review brings new inspiration for further research in this active and important topic of medical relevance.

## Figures and Tables

**Table 1:** Selected examples of biogenic metal-based NPs with antimicrobial activity synthesized by different biological sources. According to source: ^1^ = Oomycete, ^2^ = Cyanobacterium, ^3^ = Fungi, ^4^ = Plant, ^5^ = Algae, ^6^ = Bacterial, ^7^ = Microalgae.

Metal	Biocidal Activity	Biological Source for the Synthesis	Size and Shape	Characterization	Antimicrobial Dose	Reference
Ag-NP	Bactericidal	*Pythium oligandrum* ^1^	Size: 6–12 nm Shape: round	UV–Vis; TEM; XRD	0.088 mg L^−1^ 0.176 mg L^−1^ 0.44 mg L^−1^	[59]
		*Leptolyngbya* sp. WUC 59 ^2^	Size: 20–35 nm	UV–Vis; XRD; FTIR; TEM; EDXRF	10 mg L^−1^	[39]
		*Fusarium scirpi* ^3^	Size: 2–20 nm Shape: quasi-spherical	UV–Vis; FTIR; TEM; EDXRF	76 mg L^−1^	[60]
		*Calligonum comosum* ^4^ (roots) *Azadirachta indica* ^4^ (leaf extracts)	Size: 90–183 nm Shape: spherical and aggregate	FTIR; TEM; SEM	10.9–21.4 l μg mL^−1^	[61]
		*Allium sativum* ^4^	Size: 13.13–22.69 nm Shape: spherical and aggregated	UV–Vis; SEM; FTIR	30 mg mL^−1^	[62]
		*Trichodesmium erythraeum* ^7^	Size: 26.5 nm Shape: cubical	TEM; XRD; SEM; FTIR; AFM	MIC: 50–75 µg mL^−1^	[63]
	Virucidal	*Panax ginseng* (roots) ^4^	Size: 5–15 nm Shape: Spherical	UV–Vis; XRD; FTIR;TEM	0.005 M 0.01 M 0.15 M	[64]
		*Lampranthus* *coccineus* ^4^ *Malephora lutea* ^4^	Size: 10.12–27.89 nm (*L. coccineus*) 8.91–14.48 nm (*M. lutea*) Shape: Spherical	TEM; UV–Vis; FTIR; SEM, AFM, EDX; XRD	IC50: 29.04–31.38 µg mL^−1^	[33]
Au-NPs	Bactericidal	*Tinospora cordifolia* ^4^	Size: 16.1 nm Shape: spherical and polydisperse	UV–Vis; FTIR; XRD; EDX; SEM; TEM.	1000 µg mL^−1^	[65]
		*Codonopsis pilosula* ^4^	Size: 20 nm Shape: spherical	FTIR; XRD; TEM; EDX.		[66]
		*Mangifera indica* ^4^	Size: 46.8 nm Shape: spherical	SEM; TEM; UV-Vis; XRD; EDX.	25–100 μg mL^−1^	[67]
	Virucidal	*Allium sativa* ^4^ (garlic extract)	Size: 6 nm Shape: Spherical	UV–Vis; DLS; TEM.	EC50: 8.829 µg mL^−1^	[68]
		*Oscillatoria* sp. ^5^ *Spirulina platensis* ^5^	Size: 15.60–77.13 nm. Shape: Octahe- dral, pentagonal and triangular structures	UV–Vis; XRD; TEM; FTIR.	31.25 μL well^−1^	[30]
Cu-NPs CuO-NPs	Bactericidal	*Cissus vitiginea* ^4^	Size: 20 nm Shape: monodispersed distribution	UV–Vis; SEM; XRD; TEM.	25, 50 and 75 μL	[69]
		*Hagenia abyssinica (Brace) JF. Gmel.* ^4^	Size: 34.76 nm Shape: mix of spherical, hexagonal, triangular, cylindrical, and irregularly particles	UV–Vis; FTIR; XRD; TEM; EDXRF.	1 mg mL^−1^ extract	[70]
		*Curcuma longa* ^4^	Size: 5–20 nm Shape:	FESEM; SEM; TEM; XRD; EDXRD.	100–250 μL	[71]
		*Garcinia mangostana* ^4^	Size: 20–25 nm Shape: spherical and agglomerated	XRD; TEM; SEM	0.4–0.4 μg mL^−1^	[72]
	Virucidal	*Syzygium* *Alternifolium*^4^ (Fruit extract)	Size: 2–69 nm Shape: spherical and agglomerated	-	-	[73]
ZnO-NPs	Bactericidal	*Cassia fistula* ^4^ *Melia azadarach* ^4^	Size: 3–68 nm Shape: spherical	XRD; FTIR; SEM; UV-Vis; DLS.	50 µg mL^−1^ (10 µL) to 1000 µg mL^−1^ (200 µL)	[74]
		*Banana peel extract* ^4^	Size: 450 × 24 nm, 210 × 120 nm, 20–40 nm 430 × 180 nm. Shape: flakes, nanocones, pinecone-like structure and cubic.	FTIR; GPC; XRD; SEM; TGA; QMS	250 μg mL^−1^	[75]
		*Punica granatum* ^4^	Size: 50.95–54.84 nm Shape: polydispersity of nanoparticles with spikes on the surface, irregular form	XRD; UV-Vis; TEM; FTIR; EDXRD.	5000 µg mL^−1^	[76]
		Orange fruit peel ^4^	Size: 10–20 nm Shape: small and spherical	XRD; TGA; TEM.	0.025 mg mL^−1^	[66]
		*Magnoliae officinalis* ^4^	Size: 150 nm Shape: spherical	UV, FTIR, SEM, XRD, EDX, DLS	250 µg mL^−1^	[77]
		*Matricaria chamomilla* L. ^4^ *Olea europea* ^4^ *Lycopersicon esculentum* M. ^4^	Size: 48.2 nm Shape: cubic structures	UV–Vis; XRD; TEM; SEM.	4–16 µg mL^−1^	[78]
		*Catharanthus roseus* ^4^	Size: 50.73 nm Shape: spherical	UV–Vis; FTIR; XRD; TEM; SEM; EDX; AFM; DLS.	1500 µg mL^−1^	[79]
	Virucidal	-	-	-	-	-
TiO_2_-NPs	Bactericidal	*Tricoderma citrinoviride* ^3^	Size: 10–400 nm Shape: irregular, triangular, pentagonal, spherical and rod-shaped particles	UV–Vis; FESEM; SEM; FTIR; XRD; DLS.	50–100 µg mL^−1^	[80]
		*Staphylococcus aureus* ^6^	Size: 10–30 nm Shape: spherical, oval, and smooth surface	UV–Vis; XRD; SEM; FTIR; AFM;	10–15 mg mL^−1^	[81]
		*Cola nitida* ^4^	Size: 25–191 nm Shape: spherical	UV–Vis; FTIR; TEM; EDX.	80 µg mL^−1^	[82]
	Virucidal	*-*	-	-	-	-
FeO-NPs	Bactericidal	*Pterocladia capillacea* ^4^	Size: 11.24–33.71 nm Shape: nanospheres	FTIR; SEM; EDXRD.	30 mg mL^−1^	[54]
		*Agrewia optiva* ^4^ *Prunus pérsica*^4^	Size: 15–60 nm Shape: rough surfaces, agglomerated, quasi-spherical	UV–Vis; FTIR; XRD; SEM; TEM; DLS.	100 Μl NPs	[83]
		*Zingiber officinale k* ^4^	Size: 56.2 nm Shape: agglomerated and larger	UV–Vis; FTIR; XRD	30 μg mL^−1^	[84]
		*Psidium guajava* ^4^	Size: 34 nm Shape: quasi-spherical	XRD; UV–Vis; FTIR; SEM; TEM; VSM.	20–100 μg mL^−1^	[85]
	Virucidal	*-*	-	-	-	-

EDXRF: Energy-dispersive X-ray spectroscopy; TEM: transmission electron microscopy; FTIR: Fourier transform infrared; XRD: X-ray diffraction; UV–Vis: UV–Vis spectroscopy; AFM; Atomic Force Microscopy; SEM: scanning electron microscopy; DLS: Dynamic light scattering; FESEM: Field emission scanning electron microscopy; GPS: gel permeation chromatography; QMS: quadrupole mass spectroscopy; VSM: vibrating-sample magnetometer.
